# Long‐term microbial community dynamics at two full‐scale biotrickling filters treating pig house exhaust air

**DOI:** 10.1111/1751-7915.13417

**Published:** 2019-05-20

**Authors:** Caroline Van der Heyden, Thijs De Mulder, Eveline I. P. Volcke, Peter Demeyer, Marc Heyndrickx, Geertui Rasschaert

**Affiliations:** ^1^ Department of Biosystems Engineering Ghent University Coupure links 653 9000 Gent Belgium; ^2^ Technology and Food Science Unit Flanders Research Institute for Agriculture Fisheries and Food (ILVO) Burgemeester Van Gansberghelaan 115, bus 1 9820 Merelbeke Belgium; ^3^ Technology and Food Science Unit Flanders Research Institute for Agriculture Fisheries and Food (ILVO) Brusselsesteenweg 370 9090 Melle Belgium; ^4^ Department of Pathology, Bacteriology and Avian Diseases Faculty of Veterinary Medicine Ghent University Salisburylaan 133 9820 Merelbeke Belgium

## Abstract

In this study, the microbial community structure of two full‐scale biotrickling filters treating exhaust air from a pig housing facility were evaluated using 16S metabarcoding. The effect of inoculation with activated sludge of a nearby domestic waste water treatment plant was investigated, which is a cheap procedure and easy to apply in practice. The study was performed at a three‐stage and a two‐stage full‐scale biotrickling filter; of which, only the latter was inoculated. Both biotrickling filters evolved towards a rather similar community over time, which differed from the one in the activated sludge used for inoculation. However, the bacterial population at both biotrickling filters showed small differences on the family level. A large population of heterotrophic bacteria, including denitrifying bacteria, was present in both biotrickling filters. In the non‐inoculated biotrickling filter, nitrite‐oxidizing bacteria (NOB) could not be detected, which corresponded with the incomplete nitrification leading to high nitrite accumulation observed in this system. Inoculation with the wide spectrum inoculum activated sludge had in this study a positive effect on the biotrickling filter performance (higher ammonia removal and lower nitrous oxide production). It could thus be beneficial to inoculate biotrickling filters in order to enrich NOB at the start‐up, making it easier to keep the free nitrous acid concentration low enough to not be inhibited by it.

## Introduction

Pig production has intensified significantly during the last decades, resulting in potentially higher emissions of pollutants from pig housing facilities. Pig exhaust air contains a high nitrogen load, mostly as ammonia, and many odorous organic compounds, which are usually low in concentration but can have a significant impact on odour nuisance (BREF, 2017). Biofiltration of air with biotrickling filters (or biological air scrubbers) is frequently used as an effective technique for exhaust treatment of mechanically ventilated animal houses (Melse and Ogink, 2005). Intense contact between the polluted air and washing water over an inert packing material drives the water‐soluble components from the gas phase to the liquid phase, thus cleaning the exhaust air.

Ammonia and other pollutants, like volatile organic compounds (VOCs), are taken up in the scrubber system and oxidized by bacteria. The microbial population of the biofilm is considered to be a consortium of bacteria, including nitrifiers and organoheterotrophs (Juhler *et al*., 2009). Nitrification mainly involves two phylogenetically unrelated groups of autotrophic bacteria, i.e. ammonia oxidizing bacteria (AOB), which convert the absorbed ammonium (${\text{NH}}_{4}^{+}$) into nitrite (${\text{NO}}_{2}^{-}$) and nitrite‐oxidizing bacteria (NOB), which convert nitrite further into nitrate (${\text{NO}}_{3}^{-}$) (Ge *et al*., 2015).

Only a few studies on the microbial community in biotrickling filters treating pig exhaust air are available (Juhler *et al*., 2009; Kristiansen *et al*., 2011a,b; Blázquez *et al*., 2017), using different analysing techniques. It has been shown using Quantitative fluorescence *in situ* hybridization (FISH) and through tag‐encoded FLX amplicon pyrosequencing, that heterotrophic bacteria comprise a large part of the biofilm and that nitrifying bacteria represent only a small fraction with < 5% (Kristiansen *et al*., 2011b; Blázquez *et al*., 2017). Additionally, NOB often appear to be absent, resulting in nitrite accumulation (Juhler *et al*., 2009).

Inoculation of biotrickling filters with activated sludge of a wastewater treatment plant is sometimes applied in practice in Flanders and the Netherlands. However, scientific results of the effects on microbial community are lacking.

In this study, the microbial community structure of two biotrickling filters treating pig exhaust air were evaluated over time and space, additionally investigating the effect of inoculation on the microbial population. The study was performed with both a three‐stage and a two‐stage full‐scale biotrickling filter system treating pig house exhaust air. Only the two‐stage biotrickling filter was inoculated with thickened activated sludge of a nearby domestic wastewater treatment plant. The bacterial population was monitored during the first 3 months of the start‐up using 16S metabarcoding of samples collected at the different stages of the biotrickling filters and in both the biofilm and washing water.

## Experimental procedures

### Description of the biotrickling filters

**Figure 1:**
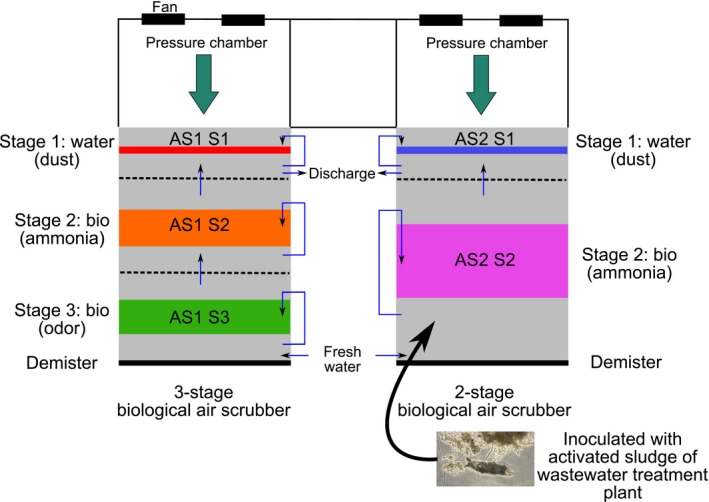
Schematic representation of the two biotrickling filters in top view.

The study was conducted at two biotrickling filters installed at one fattening pig housing facility, located in Flanders, Belgium. Biotrickling filters 1 and 2 are a three‐stage and a two‐stage crosscurrent biotrickling filter respectively (Fig. 1, see Table S1 for design specifications in supporting information). Both have the same empty bed residence time (EBRT). Both first stages, closest to the housing facility, are the dust sections with water being sprayed on top of the packing and from the front to remove as much dust from the exhaust air as possible. The second stages are called the bio sections. It is assumed that most ammonia is absorbed here. The third stage, only present at biotrickling filter 1, is generally referred to as the odour section as it is assumed that more odour treating bacteria can survive in this section. Electrical conductivity (EC) is monitored continuously with an EC sensor (ECDIND PT, Emec, Vazia, Italy) as a measure of total nitrogen in the washing water. At a threshold value of about 10–20 mS cm^−1^, corresponding to 2.3–5.1 gN l^−1^, the washing water is discharged from the first stage (dust section) into a tank. Freshwater is then added at the last stage of each biotrickling filter (bio or odour section respectively) to replace the discharged and evaporated washing water. The water thus flows in the opposite direction as that of the airflow, allowing the dirtiest washing water to come into contact with the dirtiest air and the cleanest water with the cleanest air, increasing the driving force of the latter. Consequently, the concentration of the pollutants increases in the washing water, from stage 3 to stage 1. The discharge rate measured on average 0.44 m^3^ day^−1^ for biotrickling filter 1 and 0.30 m^3^ day^−1^ for biotrickling filter. Therefore, the hydraulic retention time (HRT) of the water in the buffer tank was, respectively, 7.3 and 7.7 days, meaning it took approximately 7 days before all water was refreshed in the buffer tank or 13% of the buffer tank was refreshed per day. Most biological air scrubbers installed at pig housing facilities have a comparable HRT.

Both biotrickling filters were started up in March 2016 and were fully operating in April 2016. Biotrickling filter 1 was not inoculated. Biotrickling filter 2 was inoculated twice at 4 April and 12 September 2016, with approximately 1 m^3^ thickened activated sludge water from a nearby domestic wastewater facility in a buffer tank of 4 m³. Before the second inoculation, the last buffer tanks of both biotrickling filters were emptied and refilled with freshwater. Continuous measurements of the performance of both biotrickling filters with regard to ammonia removal and nitrous oxide production were carried out between April 2016 and March 2017, and the results are reported (C. Van der Heyden, E.I.P. Volcke, E. Brusselman and P. Demeyer, submitted). An overview of the performance and most important operational parameters are summarized in Table 1. Both biotrickling filters performed well for ammonia removal after start‐up, but the two‐stage inoculated biotrickling filter showed the highest and most stable ammonia removal over time. The average ammonia removal efficiency was 50.5 ± 29.7% for biotrickling filter 1, which was significantly lower (*P* < 0.0001) than the removal efficiency of biotrickling filter 2 (70.0 ± 27.4%). Nitrous oxide was produced in both biotrickling filters. The production of nitrous oxide varied considerably during the entire measuring period for both scrubbers. The average nitrous oxide production over the entire measuring period was 39.1 ± 28.5% for the three‐stage air scrubber and 31.0 ± 23.9% for the two‐stage air scrubber. Taking into account only the last period, from January 2017 onwards, when the ammonia removal efficiency reached a more constant level, the nitrous oxide production measured 48.3 ± 28.3% at biotrickling filter 1, slightly higher than at biotrickling filter 2, 29.2 ± 17.9% (*P* < 0.0001). When the nitrous oxide production is expressed as the produced nitrous oxide concentration compared with the incoming ammonia concentration in the biotrickling filter, the average of the three‐stage air scrubber amounts 5.3 ± 1.4% and of the two‐stage biotrickling filter 4.7 ± 1.5%. This is within the boundaries found in other biotrickling filters, ranging around 3% up to 66% (Melse *et al*., 2012; Van der Heyden *et al*., 2016). The three‐stage non‐inoculated biotrickling filter suffered from incomplete nitrification characterized by nitrite accumulation. The free ammonia (FA, NH_3_) and free nitrous acid (FNA, HNO_2_) concentration in both air scrubbers, exceeded the boundary for inhibition of NOB, for at least a couple of periods, which could explain the observed nitrite accumulation.

**Table 1:** Overview the biotrickling filter performances and operational parameters (average values over period April 2016 till March 2017 or ranges)

	Biotrickling filter 1 3‐stage non‐inoculated	Biotrickling filter 2 2‐stage inoculated
NH_3_ removal (%)	50.51 ± 29.73	70.02 ± 27.43
N_2_O production (%)	39.1 ± 28.5	31.0 ± 23.9
pH (−)	7.5 ± 0.6	7.1 ± 0.6
EC (mS cm^−1^)	18.7 ± 13.0	20.6 ± 11.3
${\text{NH}}_{4}^{+}$ (gN l^−1^)	2.5 ± 1.9	2.8 ± 1.8
${\text{NO}}_{2}^{-}$ (gN l^−1^)	2.1 ± 1.5	0.6 ± 0.8
${\text{NO}}_{3}^{-}$ (gN l^−1^)	0.5 ± 0.3	1.8 ± 1.3
FA (mgN l^−1^)	0–119	0–90
FNA (mgN l^−1^)	0–1.96	0–0.66

### Sample collection

Three sample types were collected: from the activated sludge used as the inoculum, from the biofilm and from the washing water. At the two moments of inoculation, i.e. day 1 (April 4th) and day 162 (September 12th), activated sludge samples (SL) were collected. In the biotrickling filter, biofilm (B) and washing water (W) samples were collected from each stage at day 19 (April 22nd), day 73 (June 15th), day 185 (October 5th) and day 227 (November 16th). Composite biofilm samples were collected from various positions over the entire surface area of the packing. Washing water was sampled in the top layer (max 5 cm deep). All samples (Table 2) were collected in sterile 15 ml tubes and stored at −20°C.

**Table 2:**
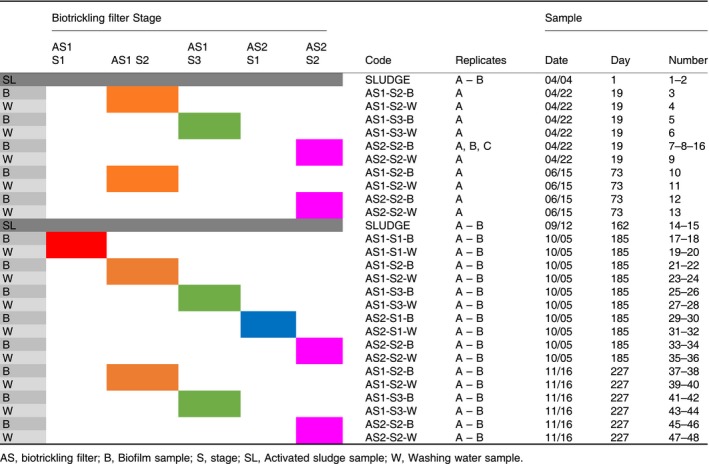
Overview of samples collected at different days and locations in the two biotrickling filters. Colour codes correspond to the positions in the biotrickling filters as in Fig. 1

### DNA extraction and 16S Amplicon Sequencing

DNA extraction was carried out using the Powersoil DNA Isolation Kit (MOBIO Laboratories, Carlsbad, CA, USA) according to the manufacturer's instructions, using 250 mg of biofilm, pellet from washing water or activated sludge. The washing water samples were centrifuged at 10 000 rpm for 1 min. The precipitate was scraped off and if not enough was present, supernatant was used to reach 250 mg sample. DNA quantity and quality was measured using the NanoDrop ND‐1000 (Thermo Scientific, Wilmington, NC, USA) and the Quantus double‐stranded DNA assay (Promega, Madison, WI, USA).

The taxonomic profiles of bacterial communities were determined using amplicon sequencing of the V3–V4 variable region of the 16S rRNA gene. The library preparation was based on the Illumina 16S metagenomic sequencing library preparation protocol (Illumina, 2013; De Mulder *et al*., 2016). The amplicon PCR was performed with the primers described by Klindworth *et al*. (2013). The final barcoded library was sequenced using Illumina MiSeq V3‐technology (2 × 300 bp, paired‐end) by Oklahoma Medical Research Foundation (Oklahoma City, OK, USA).

### Processing of the sequence reads

The amplicon sequencing data set was demultiplexed by the sequencing provider, and barcodes were clipped off the reads. The raw sequence data are accessible from the NCBI Short Read Archive (accession number SRP152501). Primers were removed using Trimmomatic v0.32 (Bolger *et al*., 2014). Different programs of the USEARCH software v7.0.1090 were used for the following steps, in combination with software packages PEAR and QIIME. Forward and reverse reads were merged using a minimum overlap length of 120 bp, a minimum and maximum resulting length of 400 and 450 bp and a quality threshold of 30 with a minimum length of 200 bp after trimming, using PEAR 0.9.8 (Zhang *et al*., 2014). The resulting sequences were quality filtered using ‘fastq_filter’ with a maximum expected error of 3. Next, sequences of all samples to be compared were merged, dereplicated (‘derep_fulllength’) and sorted by abundance (‘sortbysize’). UPARSE (‘cluster_otus’) was used for clustering the reads into operational taxonomic units (OTUs) at 97% identity level (Edgar, 2013). Chimeras were removed using UCHIME (‘uchime_ref’) with the RDP Gold database as a reference (Edgar *et al*., 2011). Finally, sequences of individual samples were mapped back to the representative OTUs using the ‘usearch_global’ algorithm (97% identity) and converted to an OTU table using ‘biom convert’ (McDonald *et al*., 2012). This procedure resulted in an average of 47 581 sequences per sample with an average length of 416 bp (51 samples). Taxonomy assignment was performed using the QIIME software package with the SILVA 123 reference set and a taxonomy level of 97%.

### Downstream data analysis and statistics

The composition and structure of the bacterial communities of the different sample types (activated sludge, biofilm, washing water) were analysed. Rarefaction analysis was done using the R package Vegan (Oksanen *et al*., 2015). Samples were retained if rarefaction curves indicated that the sequencing depth was sufficient (stationary phase was reached). The alpha diversity was investigated by calculating the total number of observed species (rarefaction analysis) and estimating the diversity (Shannon−Wiener diversity index) using the Phyloseq package in R. For subsequent analysis of the beta diversity, only OTUs representing at least 0.01% of the total community in at least one sample were retained. Multivariate analysis of the data set was done as previously described (De Tender *et al*., 2015), using the R package Vegan. Differences were visualized by constructing non‐metric multidimensional scaling (nMDS) plots, using Bray−Curtis dissimilarity indices. The betadisper function was used to test the multivariate spread of the data before the significant differences between airscrubbers, sample types and sample dates were identified by permutational multivariate analyses of variance (PERMANOVA) using the Adonis and the pairwise Adonis function (package Vegan).

## Results and discussion

### Bacterial community structure

The bacterial community structure of the biotrickling filter samples was investigated. At 10 000 sequence counts, the rarefaction curves showed an average of 867, 279 and 198 different OTUs for the activated sludge, the biofilm and the washing water samples respectively. Sample number 5, 16 and 44 were excluded from analysis as not enough reads were found.

**Figure 2:**
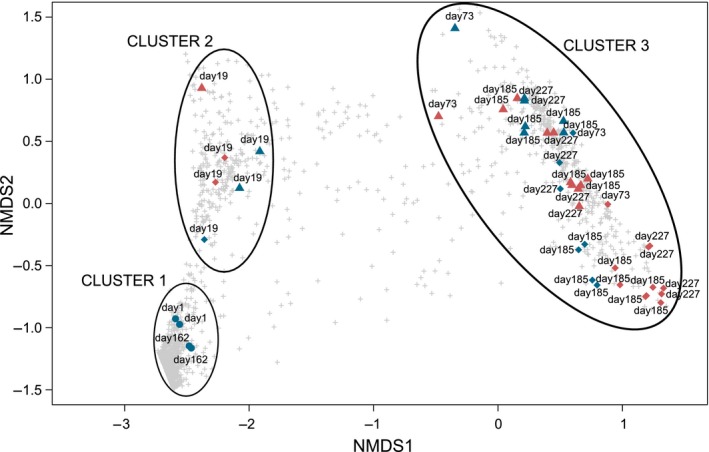
Non‐metric multidimensional scaling (nMDS) of pairwise community dissimilarity (Bray−Curtis) indices of 16S sequencing data of all samples. Shape indicates different sampling type: ▲ biofilm, ♦ washing water and ● sludge. Samples of biotrickling filter 1 are indicated in red and of biotrickling filter 2 in blue. The sample date is present at each data point. OTUs are indicated in grey (+).

Statistical analysis revealed that the microbial composition of airscrubber 1 and airscrubber 2 were significantly different from each other (*P* < 0.001). NMDS ordination of the Bray–Curtis distances of all samples demonstrated separated clustering based on collection date and sample origin (Fig. 2). The activated sludge samples clearly make up a separate cluster (cluster 1), indicating that these samples are different in richness and community composition compared with the biofilm and washing water samples (*P*_adj_ = 0.003). Two other main clusters were visually distinguished (cluster 2 and 3), indicating a possible dominant influence of sample collection moment on the microbial community composition, although this could not be further statistically analysed as the homoscedasticity assumptions were not fulfilled for sample date when all sample dates were considered. Cluster 2 contains the samples from the first sampling at day 19 of both biotrickling filters. Given their position on the plot, these samples were different from the activated sludge samples but still more related to the activated sludge samples than to the samples in cluster 3. Cluster 3 contains samples of biofilm and washing water of both biotrickling filters at sampling day 73 till 227. Its position on the plot relative to the other two clusters indicates that the biotrickling filter bacterial communities evolved further away from those of the activated sludge samples. However, cluster 3 comprises samples from both biotrickling filter 1 and 2, without a clear separation in the overall nMDS plot (Fig. 2), although biotrickling filter 2 was inoculated whereas biotrickling filter 2 was not.

**Figure 3:**
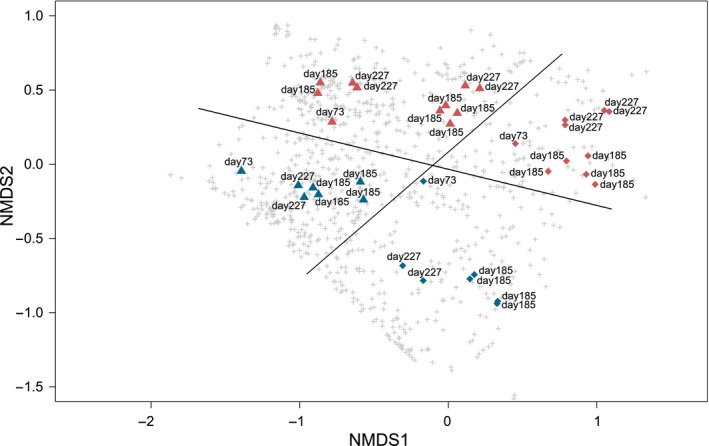
Non‐metric multidimensional scaling (nMDS) of pairwise community dissimilarity (Bray−Curtis) indices of 16S sequencing data of the samples of the last sampling period (cluster 3). Shape indicates different sampling type: ▲ biofilm and ♦ washing water. Samples of biotrickling filter 1 are indicated in red and of biotrickling filter 2 in blue. The sample date is indicated at each data point. OTUs are indicated in grey (+).

If only the samples in cluster 3 are ordinated with nMDS, a more profound distinction, without any observed overlap, can be made between the two biotrickling filters (Fig. 3). Indeed, without the sample days 1 and 162 (sludge samples) and day 19, the airscrubbers were significantly different (*P* < 0.001). Further, day 73 was significantly different from day 185 (*P*_adj_ = 0.015), whereas there was no significant difference between day 185 and 227 nor between day 73 and 227.

### Composition of the bacterial communities

In the data set, a total of 38 phyla and 342 families were identified across all samples. An overview of the bacterial community structure during the two last sampling days (185 and 227), per biotrickling filter, stage and sample type, is provided in Table 3. OTUs that could not be assigned to a specific taxon were grouped under ‘Unassigned’. On average, 1% of the OTUs could not be identified to family level.

**Table 3:** Overview of the average relative abundance (%) and standard deviation of the major bacterial phyla and major families, discussed in this study. Sampling days 73 and 162 for the activated sludge samples and 185 and/or 227 for the biotrickling filter samples were taken into account. A zero means that in none of those samples, OTUs of this phylum or family were found

Taxonomy	Activated sludge	Biotrickling filter 1 – non‐inoculated						Biotrickling filter 2 – inoculated			
		Stage1		Stage 2		Stage 3		Stage 1		Stage 2	
		Biofilm	Water	Biofilm	Water	Biofilm	Water	Biofilm	Water	Biofilm	Water
Sampling day	73 + 162	185	185	185 + 227	185 + 227	185 + 227	185 + 227	185	185	185 + 227	185 + 227
*Proteobacteria*	40.1 ± 1.1	39.7 ± 0.5	50.8 ± 2.8	45.2 ± 2.8	51.2 ± 7.4	57.7 ± 8.8	38.0 ± 3.9	42.5 ± 0.4	24.7 ± 0.1	51.6 ± 2.2	26.3 ± 15.3
*Comamonadaceae*	11.2 ± 5.1	17.0 ± 0.9	43.6 ± 4.3	22.8 ± 10.1	39.5 ± 3.5	27.9 ± 4.6	34.3 ± 4.6	14.4 ± 0.1	6.3 ± 0.5	14.8 ± 2.4	4.6 ± 1.0
*Xanthomonadaceae*	3.4 ± 0.8	5.5 ± 0.02	1.4 ± 0.3	6.7 ± 4.3	0.4 ± 0.2	14.2 ± 2.0	0.5 ± 0.2	14.0 ± 0.7	2.8 ± 0.2	17.9 ± 3.6	2.8 ± 2.8
*Alcaligenaceae*	0.4 ± 0.2	6.4 ± 1.4	2.4 ± 0.1	4.7 ± 2.9	2.2 ± 0.6	0.9 ± 0.3	0.5 ± 0.3	4.6 ± 0.2	6.8 ± 0.2	6.2 ± 3.3	8.7 ± 6.8
*Rhodocyclaceae*	5.0 ± 1.7	< 0.1	< 0.1	< 0.1	0	< 0.1	< 0.1	< 0.1	< 0.1	< 0.1	< 0.1
*Nitrosomonadaceae*	4.1 ± 0.1	0.8 ± 0.009	0.1 ± 0.02	1.5 ± 0.9	0.1 ± 0.1	10.7 ± 5.5	1.6 ± 1.1	0.6 ± 0.1	0.1 ± 0.04	1.2 ± 0.1	0.3 ± 0.3
*Piscirickettsiaceae*	< 0.1	2.7 ± 0.7	1.4 ± 0.8	2.4 ± 3.2	3.7 ± 4.0	0.5 ± 0.3	0.1 ± 0.1	4.0 ± 0.5	3.6 ± 0.9	5.1 ± 1.5	4.5 ± 2.6
*Methylophilaceae*	< 0.1	1.8 ± 0.3	0.4 ± 0.03	2.5 ± 1.6	2.0 ± 2.2	0.3 ± 0.2	< 0.1	0.1 ± 0.01	0.1 ± 0.05	< 0.1	< 0.1
*Bradyrhizobiaceae*	0.6 ± 0.3	< 0.1	< 0.1	< 0.1	< 0.1	< 0.1	< 0.1	< 0.1	< 0.1	1.2 ± 0.5	< 0.1
*Legionellaceae*	< 0.1	0.1 ± 0.002	0.2 ± 0.02	< 0.1	0.2 ± 0.2	0.3 ± 0.2	0.2 ± 0.1	0.3 ± 0.1	< 0.1	0.3 ± 0.3	0.1 ± 0.1
*Bacteriodetes*	29.1 ± 0.5	45.0 ± 0.2	27.5 ± 1.1	38.4 ± 2.6	30.0 ± 3.7	21.6 ± 5.7	44.7 ± 2.4	33.0 ± 2.4	35.9 ± 0.5	29.0 ± 3.6	35.7 ± 10.1
*Flavobacteriaceae*	0.6 ± 0.5	30.6 ± 0.2	16.2 ± 0.1	28.5 ± 2.5	20.2 ± 5.4	6.8 ± 4.8	32.9 ± 4.2	17.7 ± 1.9	7.5 ± 0.2	13.4 ± 5	8.9 ± 5.3
*Cytophagaceae*	0.2 ± 0.1	1.7 ± 0.1	6.6 ± 1.7	0.9 ± 0.9	6.1 ± 3.8	0.6 ± 0.3	10.4 ± 2.7	1.7 ± 0.5	20.9 ± 0.1	0.7 ± 0.5	15.6 ± 18.2
*Chitinophagaceae*	10.4 ± 4.1	9.2 ± 0.3	3.2 ± 0.3	5.4 ± 1.3	2.7 ± 0.3	7.8 ± 1.2	0.6 ± 0.2	9.3 ± 1.2	3.5 ± 0.5	9.5 ± 1.8	6.6 ± 3.3
*Cryomorphaceae*	0.1 ± 0.1	1.5 ± 0.05	0.5 ± 0.02	1.6 ± 0.6	0.6 ± 0.2	1.7 ± 0.9	0.4 ± 0.1	1.2 ± 0.3	3 ± 0.2	0.9 ± 0.7	3.4 ± 2.0
*Saprospiraceae*	11.2 ± 4.0	0.7 ± 0.2	0.1 ± 0.001	0.6 ± 0.2	0.1 ± 0.1	0.4 ± 0.2	< 0.1	0.2 ± 0.03	0.1 ± 0.01	0.7 ± 0.5	0.1 ± 0.1
*Actinobacteria*	5.7 ± 2.4	4.9 ± 0.01	12.2 ± 4.0	5.6 ± 2.1	8.4 ± 7.1	8.4 ± 2.1	6.9 ± 4.4	8.6 ± 1.8	22.8 ± 0.9	6.8 ± 3.4	20.6 ± 14.3
*Intrasporangiaceae*	0.2 ± 0.1	1.4 ± 0.1	11.5 ± 3.8	1.8 ± 0.8	7.9 ± 7.4	2.5 ± 1.0	6.7 ± 4.4	3.2 ± 1.7	20.5 ± 1.3	3.5 ± 3.1	19.1 ± 14.5
*Saccharibacteria*	4.5 ± 0.5	2.5 ± 0.2	6.9 ± 0.5	2.3 ± 0.9	6.5 ± 4.7	4.0 ± 1.1	7.9 ± 3.8	2.3 ± 0.1	8.8 ± 0.2	2.1 ± 0.4	10.3 ± 2.5
*Deinococcus–Thermus*	< 0.1	2.4 ± 0.4	0.4 ± 0.2	4.1 ± 1.2	0.1 ± 0.1	1.2 ± 0.4	< 0.1	5.4 ± 0.2	0.3 ± 0.1	2.9 ± 0.8	0.1 ± 0.2
*Trueperaceae*	< 0.1	2.4 ± 0.4	0.4 ± 0.3	4.1 ± 1.9	<0.1	1.2 ± 0.4	< 0.1	5.7 ± 0.2	0.3 ± 0.1	2.9 ± 0.8	0.1 ± 0.2
*Chloroflexi*	5.0 ± 0.7	0.1 ± 0.02	< 0.1	0.1 ± 0.04	< 0.1	0.1 ± 0.05	< 0.1	0.2 ± 0.006	< 0.1	0.2 ± 0.08	< 0.1
*Acidobacteria*	3.6 ± 1.8	0	< 0.1	< 0.1	< 0.1	0	0	< 0.1	0	0.1 ± 0.1	< 0.1
*Nitrospirae*											
*Nitrospiraceae*	0.8 ± 0.3	0	0	0	0	0	0	< 0.1	0	< 0.1	0
*Unassigned*	1.1 ± 0.3	1.9 ± 0.3	0.5 ± 0.03	1.3 ± 0.5	1.0 ± 0.8	1.8 ± 1.0	0.4 ± 0.3	1.8 ± 0.2	1.9 ± 0.2	1.7 ± 0.2	0.8 ± 0.2

The activated sludge samples showed a high relative abundancy of the phyla Proteobacteria (40%) and Bacteroidetes (29%). The other main phyla in the activated sludge samples were Actinobacteria (5.7%), Saccharibacteria (4.5%), Chloroflexi (5.0%) and Acidobacteria (3.6%). The most abundant families found in the activated sludge samples were the *Comamonadaceae* (11%) belonging to the Proteobacteria and *Saprospiraceae* (11%), *Chitinophagaceae* (10%) and *Rhodocyclaceae* (5.0%) belonging to the Bacteroidetes. The phyla Proteobacteria, Bacteroidetes and Actinobacteria accounted for more than 80% of the community in all biotrickling filter samples. Chloroflexi and Acidobacteria represented < 0.2% in the samples of the biotrickling filters, showing a difference in community with the activated sludge samples. In addition, the family of *Rhodocyclaceae* (Proteobacteria) and *Saprospiraceae* (Bacteroidetes) were not very prevalent in the biotrickling filter samples compared with the activated sludge samples.

Previous studies on biotrickling filters and biofilters treating high levels of gaseous ammonia showed similar communities, with Proteobacteria, Bacteroidetes and Actinobacteria as the dominant phyla, although differing in relative abundances (Kristiansen *et al*., 2011b; Blázquez *et al*., 2017). A common core of bacterial families was present in both biotrickling filters, although the communities differentiated between sample time and compartment in terms of relative abundances. One of the largest prevailing families in the biotrickling filter samples was *Comamonadaceae* (Proteobacteria), although its relative abundances differed from sample to sample (4.6–44%). In biotrickling filter 1, the abundance of *Comamonadaceae* was higher in the washing water samples, whereas in biotrickling filter 2, this family was found more in the biofilm samples. This family was also abundantly present in other biotrickling filters, treating pig exhaust air (Kristiansen *et al*., 2011a,b; Blázquez *et al*., 2017). It is a large and diverse bacterial family belonging to the order *Burkholderiales*, containing genera that includes aerobic organotrophs, anaerobic denitrifiers and Fe^3+^‐reducing bacteria, hydrogen oxidizers, photoautotrophic and photoheterotrophic bacteria, and even fermentative bacteria (Willems, 2014). Different genera belonging to this family were found in all samples. *Comamonas* is generally one of the most abundant microorganisms in biofilm communities driving wastewater treatment (Wu *et al*., 2015). Under bulk aerobic condition, biofilms of *C. testosteroni* were capable of reducing nitrate, which even seemed to be beneficial for the biofilm lifestyle to reduce cell detachment (Wu *et al*., 2015). As a consequence, *Comamonas* species can play a key role in denitrification under bulk aerobic conditions in biofilms (Blázquez *et al*., 2017). In our samples, the denitrifier *C. nitrativorans* was abundantly present, which is known for the ability to perform anoxic reduction of nitrate, nitrite and nitrous oxide to nitrogen (Etchebehere *et al*., 2001). Denitrification could explain why the N balance in these two biotrickling filters could not be closed (around 30% nitrogen loss).

According to a previous study at biotrickling filters (Kristiansen *et al*., 2011b), *Comamonadacea* are mainly responsible for the removal of highly soluble and easy degradable compounds as they are also known to utilize a variety of volatile fatty acids (VFA) and aromatic compounds under aerobic and denitrifying conditions. The aerobic activity was strongly dominated by heterotrophs, which accounted for 73–100% of the total oxygen uptake rate in the biofilm (Juhler *et al*., 2009). It can thus be concluded that considerable amounts of heterotrophic families were present in the investigated biotrickling filters.

The families *Flavobacteriaceae*,* Alcaligenaceae*,* Cytophagaceae*,* Cryomorphaceae*,* Piscirickettsiaceae* and *Trueperaceae* were present in some biotrickling filter samples, including the inoculated biotrickling filter 2, but were low abundant to nearly absent in the activated sludge samples. The family of *Trueperaceae* is known to be capable of resisting great environmental hazards and to reside in wastewater (Griffiths and Gupta, 2007). It was furthermore found in high‐strength ammonium wastewater, for example originating from landfill leachate (Tan *et al*., 2008; Miao *et al*., 2016). The family *Legionellaceae* was also present in some of the biotrickling filter samples at low abundancy (< 0.5%). The presence of the pathogen *Legionalla* was found in other studies (Kristiansen *et al*., 2011b; Blázquez *et al*., 2017) but not considered to be alarming (Melse *et al*., 2015). According to other authors (Kristiansen *et al*., 2011b), the biofilm of a biotrickling filter treating pig house air contain a specialized bacterial community at family level adapted to the unique extreme conditions in these biofilms, ensuring that microorganisms originating from pig faeces, as well as pathogens, such as species of *Clostridium*,* Mycobacterium* or *Legionella*, are unable to establish in the biofilm due to a strong selective pressure.

Comparing the biofilm with the water samples, it appeared that in both sample types, the phyla Proteobacteria, Bacteroidetes and Actinobacteria were the most abundant. Biotrickling filter 2 showed a higher relative abundancy of Bacteroidetes and Actinobacteria, but a lower abundancy of Proteobacteria in the washing water samples compared with the biofilm samples. Additionally, a clear difference in abundancy for the phylum Saccharibacteria was present between the biofilm and water samples, with a higher abundancy in the water samples. The biofilm samples had a higher abundance of the phyla Deinococcus–Thermus (3.3%). The latter was < 0.2% represented in the water samples and < 0.1% in activated sludge sample. The families *Xanthomonadaceae* (Proteobacteria) and *Chitinophagaceae* (Bacteroidetes) were more prevailing in the biofilm and activated sludge samples, compared with the washing water samples. *Xanthomonadaceae* are abundant heterotrophs in water treatment systems that produce exopolymeric substances (EPS) involved in biofilm formation and *Chitinophagaceae* degrade a wide range of polysaccharides in biofilms (Dworkin *et al*., 2006; Pal *et al*., 2012). The families *Cytophagaceae* and *Intrasporangiaceae* were detected more in the washing water samples than in the biofilm samples. Nevertheless, no taxonomic groups were unique for the biofilm or washing water environment. This could be expected as solid‐adherent bacteria can end up in the washing water due to biofilm detachment. The difference in abundances between taxa in the water and the biofilm could be because one taxon simply has a higher growth rate and is rinsed away in higher numbers, ending up in the water phase.

When focusing on the difference in biofilm between stages of each biotrickling filter, it can be noted that the abundance of Proteobacteria increases from stage 1 to stage 2 and further to stage 3. This can be attributed to *Comamonadaceae* and *Xanthomonadaceae*. The phylum Bacteroidetes seems to decrease over the different stages. In a study on a two‐stage biotrickling filter with cellulose pads (Kristiansen *et al*., 2011b), a small difference between both stages was reported as well. In the first stage of that study, Bacteroidetes was the most dominant group, followed by Betaproteobacteria and Actinobacteria. In the second stage, Betaproteobacteria were most commonly observed, followed by Bacteroidetes and Gammaproteobacteria.

### The nitrifying bacterial community

Ammonia oxidizing bacteria are divided into two monophyletic lineages based on their respective 16S rRNA gene sequences (Junier *et al*., 2010). The first lineage belongs to the Betaproteobacteria and comprises amongst others *Nitrosomonas* and *Nitrosospira* in the family *Nitrosomonadaceae* (Prosser *et al*., 2014). The second lineage, affiliated with the Gammaproteobacteria in the family *Chromatiaceae* (Klotz *et al*., 2006), contains *Nitrosococcus oceani* and *Nitrosococcus halophilus* (Ward and O'Mullan, 2002) amongst others. NOB are divided into four genera: *Nitrobacter* (family *Bradyrhizobiaceae;* (de Souza *et al*., 2014)), *Nitrococcus* (family *Ectothiorhodospiraceae;* Imhoff, 2014), *Nitrospira* (family *Nitrospiraceae;* (Daims, 2014)) and *Nitrospina* (family *Nitrospinaceae;* (Lücker and Daims, 2014)) from which *Nitrobacter* and *Nitrospira* are the most important for nitrification (Ge *et al*., 2015). *Nitrospira* is regarded as K‐strategist (with high substrate affinities and low maximum activity) for nitrite and oxygen, while *Nitrobacter* is an r‐strategist under limited substrate conditions (Ge *et al*., 2015).

Focusing on the nitrifying community, in particular AOB, it was observed that the family *Nitrosomonadaceae* was present in all samples (Table 3). Almost all were represented by the genus *Nitrosomonas*; however, some traces of *Nitrosospira* were also found. These findings were in agreement with previous studies which reported *Nitrosomonas and Nitrospira* as the most prevailing genera representing AOB in biotrickling filters (Juhler *et al*., 2009; Kristiansen *et al*., 2011a,b; Blázquez *et al*., 2017). In the activated sludge samples, the abundancy of *Nitrosomonadaceae* was 4.1%. In the biotrickling filter samples, their abundance varied from 0.02% in the washing water to more than 11% in some biofilm samples at both biotrickling filters. At day 185 after start‐up, a clear increase in abundance of *Nitrosomonadaceae* was observed in the biofilm from stage 1 to stage 2 and stage 3 (if present). As a result of the overall countercurrent air–water flow in the biotrickling filters, ammonia and VOC concentrations were expected to decrease from the biotrickling filter air inlet towards the outlet. Although generally the 2nd stage is referred to as the biological section for ammonia removal and the 3rd stage the odour section for odorous component removal, the results of this study show the opposite as more nitrifying bacteria were present at the last stages. This is in accordance with previous findings (Juhler *et al*., 2009; Kristiansen *et al*., 2011b), who reported a higher abundancy of AOB at the 2nd stage compared with the 1st stage. This was attributed to a mixed biofilm community of heterotrophic and nitrifying bacteria competing for space and oxygen. Due to a significantly lower growth rate and dependency on oxygen, nitrifiers can only establish persistent populations in biofilm strata where the heterotrophs are limited by substrate and not by oxygen. As the exhaust air of pig housing facilities contains a large load of VOCs, heterotrophic activity is located mainly in the first stages and consequently, the nitrifying community will only be abundant at the last stages, where most soluble organics were not present anymore. The washing water at the last stages also contained the lowest pollutant load as freshwater was added there. Strikingly, removal of ammonia from the air to the water was mainly accomplished by the first filter sections (Kristiansen *et al*., 2011a; Ottosen *et al*., 2011), where the nitrifying community was the lowest. It thus seemed that absorption was sufficient to remove ammonia from the air to the water, whereby the absorbed ammonia is slowly oxidized into nitrite and nitrate by the nitrifying population or by discharge, without a considerable effect on the ammonia removal efficiency.

The NOB containing family *Nitrospiraceae* was observed in some samples (Table 3), as well as the family *Bradyrhizobiaceae*. The genus *Nitrobacter* was not detected. Other studies showed no presence of any NOB (Kristiansen *et al*., 2011b), or only the genus *Nitrobacter* was present (Juhler *et al*., 2009; Blázquez *et al*., 2017). *Nitrospira* was present in the activated sludge samples at a low abundance of 0.80%. Interestingly, at the non‐inoculated biotrickling filter 1, NOB could not be detected in any of the samples whereas the inoculated biotrickling filter showed a low abundance of *Nitrospira* in the biofilm samples, though this differed over time. At day 19, 73, 185 and 227, the abundancy at stage 2 was, respectively, 0.3%, 0%, 0.0006% and 0%. This shows the low survival of NOB in biotrickling filters and confirms that inoculation could help to introduce NOB in the system (Juhler *et al*., 2009), as NOB could establish together with the AOB during start‐up.

### Inoculation and biotrickling filter performance

The presence of NOB in the inoculated biotrickling filter explains why it could establish full nitrification, without nitrite accumulation, while the non‐inoculated biotrickling filter, showed a considerable nitrite accumulation (Table 1). High concentrations of nitrite are known to increase nitrous oxide production (Kampschreur *et al*., 2009). The inoculated biotrickling filter had on average a lower nitrous oxide production compared with the non‐inoculated biotrickling filter (C. Van der Heyden, E.I.P. Volcke, E. Brusselman and P. Demeyer, submitted). NOB are sensitive to inhibition effects by free ammonia and free nitrous oxide. By enriching NOB at the start‐up, they immediately can convert nitrite to nitrate, keeping the free nitrous acid concentration low enough to not be inhibited by it. As inoculation with activated sludge of a wastewater treatment plant is an easy and cheap method, it could thus be beneficial to inoculate biotrickling filters in order to promote NOB, thus reducing nitrite accumulation and consequently lower the nitrous oxide production.

The nMDS plot (Fig. 2) indicates that the bacterial population of the inoculum evolves very quickly to a biotrickling filter specific microbial population. Activated sludge is a wide spectrum inoculum and thus contains a variety of bacterial families and species. In terms of ammonia removal and conversion in biotrickling filters, only the phylogenetical groups AOB, NOB and (if present) denitrifiers, are important. A specific inoculum containing only AOB and NOB could therefore also be an option to apply (Xue *et al*., 2010).

The removal of odorous components or less water‐soluble components like methane was not taken into account in this study. The operational conditions and the bacteria coming from the pig house exhaust air contribute as well to which bacteria will be selected to form a stable population. More research is necessary to investigate if an optimized inoculum would result in a better biotrickling filter performance. Additionally, this study was performed at two full‐scale biotrickling filters, during start‐up. It must be further investigated if inoculation could result in a more stable NOB population at a nitrite accumulating biotrickling filter in full operation.

## Conclusions

- The microbial population at two full‐scale biotrickling filters treating pig housing outlet air evolved towards a similar specialized community structure, despite differences in configuration (3‐stage versus 2‐stage) and the fact that only one biotrickling filter was inoculated with activated sludge. In both biotrickling filters, only a small percentage of nitrifying bacteria (AOB and NOB) and a large population of heterotrophic bacteria were present. The denitrifier *Comamonas nitrativorans* was abundantly present, confirming the possibility of denitrification in the prevailing bulk aerobic conditions.
- Different abundances of some families were observed between the biofilm and washing water samples and between the different scrubber stages. Nevertheless, no unique taxonomic groups could be distinguished between the biofilm and the washing water environment. This could be expected as solid‐adherent bacteria can end up in the washing water due to biofilm detachment. Nitrifying bacteria were more abundantly present at the last scrubber stages as they have problems of competing with the heterotrophs in the first stages.
- Inoculation with the wide spectrum inoculum activated sludge, which is a cheap procedure and easy to apply in practice, had in this study a positive effect on the biotrickling filter performance (higher ammonia removal and lower nitrous oxide production). In the inoculated biotrickling filter, NOB could be detected while this was not the case in the non‐inoculated biotrickling filter. It could thus be beneficial to inoculate biotrickling filters in order to enrich NOB at the start‐up, making it easier to keep the free nitrous acid concentration low enough to not be inhibited by it. It can be further investigated if inoculation with activated sludge could also result in a more stable NOB population at a biotrickling filter in full operation which already shows nitrite accumulating.

## Conflict of interest

None declared.

## Supporting information


**Table S1.** Design specifications of the biotrickling filters under study.
