# Supplementary material for: Long‐term microbial community dynamics at two full‐scale biotrickling filters treating pig house exhaust air
Source: Microb Biotechnol. 2019 May 20;12(4):775–86. doi: 10.1111/1751-7915.13417 (PMC6559015; doi:10.1111/1751-7915.13417)
Supplement: Supplementary file 1 — Table S1. Design specifications of the biotrickling filters under study. [file MBT2-12-775-s001.docx]

Table S1: Design specifications of the biotrickling filters under study

|  | Biotrickling filter 1  3-stage  non-inoculated | Biotrickling filter 2  2-stage  inoculated |
| --- | --- | --- |
|  | crosscurrent | crosscurrent |
| Dimensions^(1)^  [m x m x m] | 6 x 2.85 x 2.7  Stage1: 0.15  Stage2: 0.90  Stage3: 0.90 | 6 x 2.85 x 2.7  Stage1: 0.15  Stage2: 1.80 |
| Packing volume  [m^3^] | 15.0 | 15.0 |
| Buffer tank volume ^(2)^  [m^3^] | 4.1  Stage1: 1.1  Stage2: 1.5  Stage3: 1.5 | 4.1  Stage1: 1.1  Stage2: 3.0 |
| Specific surface area ^(2)^  [m^2^.m^-3^] | Stage1: 240  Stage2: 125  Stage3: 125 | Stage1: 240  Stage2: 125 |
| Maximum air flow rate  [m^3^.h^-1^] | 30720 | 30720 |
| Water flow rate ^(2)^  Recirculation rate  [m³.h^-1^] | Stage1: 20.0  Stage2: 16.5  Stage3: 19.0 | Stage1: 19.5  Stage2: 19.5 |
| Water distribution system ^(2)^ | Stage1: sprinklers  Stage2 and 3: passive distribution gutter | Stage1: sprinklers  Stage2: sprinklers |
| Minimal liquid to gas ratio  [-] | 0.0018 | 0.0012 |
| Sprinkling density  [m^3^.(m^2^.h)^-1^] | 10.0 | 7.0 |
| Minimal EBRT  [s] | 1.76 | 1.76 |
| Maximal LOAD  [m^3^.(h.m^2^)^-1^] | 3992 | 3992 |

1. Total length x width x height; Length of the three-stage and two-stage scrubber packing is given separately as well for each packing stage, with empty space between the stages.
2. Dimensions for each scrubbing stage given separately.
